# Reverse effects of *Streptococcus mutans* physiological states on neutrophil extracellular traps formation as a strategy to escape neutrophil killing

**DOI:** 10.3389/fcimb.2022.1023457

**Published:** 2022-11-10

**Authors:** Lijia Huang, Wenhua Lu, Yang Ning, Jia Liu

**Affiliations:** ^1^ Hospital of Stomatology, Guanghua School of Stomatology, Sun Yat-Sen University, Guangdong, Guangzhou, China; ^2^ Guangdong Provincial Key Laboratory of Stomatology, Sun Yat-Sen University, Guangdong, Guangzhou, China; ^3^ State Key Laboratory of Oncology in South China, Collaborative Innovation Center for Cancer Medicine, Sun Yat-sen University Cancer Center, Guangdong, Guangzhou, China; ^4^ Department of Periodontology, Guanghua School of Stomatology, Hospital of Stomatology, Sun Yat-Sen University, Guangdong, Guangzhou, China

**Keywords:** neutrophil extracellular traps (NETs), *Streptococcus mutans*, biofilm, planktonic, immune defense

## Abstract

Bacteria in nature are present in different lifestyles with distinct characteristics. *Streptococcus mutans* is the etiologic pathogen of dental caries and could easily gain access into the bloodstream after oral surgery and adopt a biofilm lifestyle, resulting in infective endocarditis. A growing amount of evidence have revealed that the large web-like structure composed of extracellular DNA and antimicrobial proteins released by neutrophils, named Neutrophil Extracellular Traps (NETs), play an active role in the defense against bacterial invasion. The present study demonstrated that NETs formation was discriminatively affected by *S. mutans* biofilm and its planktonic counterpart. The free-floating planktonic *S. mutans* exhibited an active NETs response, whereas the biofilm community exhibited a reverse negative NETs response. Besides, impaired biofilm killing correlated with the decrease in NETs production. Unlike planktonic cells, biofilm avoided the burst of reactive oxygen species (ROS) when co-culture with neutrophils, and the NADPH-oxidase pathway was partially involved. A mice infection model also supported the distinguishing response of neutrophils challenged by different lifestyles of *S. mutans.* In conclusion, different bacterial physiological states can affect the distinct response of the host–microbe interaction, thus contributing to the anti-pathogen immune response activation and immune surveillance survival.

## Introduction

Bacteria in nature adopt different lifestyles, such as planktonic, biofilm, and dispersed lifestyle. The lifespan of bacteria can be described as planktonic cells attached to biotic and abiotic surfaces and assembled to form microcolonies, which would finally differentiate into a mature biofilm structure. The continued development of biofilms results in nutrients deprivation, causing biofilm cells to start dispersing from the surface and returning back to the planktonic state (O’Toole et al., 2000; Roy et al., 2018). As the two major states of bacteria, planktonic and biofilm cells exhibit different characteristics when interacting with host cells (Svensäter et al., 2001; Huang et al., 2011). It is well known that biofilm cells are more resistant to antibiotics and host defenses than their planktonic counterparts, making biofilm-related infections difficult to eradicate and leading to persistent infections (Bjarnsholt et al., 2013; Algburi et al., 2017; Bhattacharya et al., 2018; Scharnow et al., 2019).

*Streptococcus mutans* (*S. mutans*), the well-recognized cariogenic species of dental caries, was reported to be detected in the heart valves and atheromatous plaques of atherosclerosis patients (Moreillon and Que, 2004; Krzyściak et al., 2014). Increasing evidence has shown that *S. mutans* cells could easily gain access into the bloodstream after dental surgery and adopt a biofilm lifestyle, resulting in infective endocarditis (Moreillon and Que, 2004; Abranches et al., 2011; Jung et al., 2012). Despite advancements in antibacterial therapy, the prevalence associated with invasive streptococcus biofilm has remained exceedingly high (Beceiro et al., 2013). Beyond recalcitrant to eradication, biofilms also play an important role in the spreading of infection through circulation within the host (Kaplan, 2010). Specifically, our previous study found that a nuclease of *S. mutans* facilitates biofilm dispersion and protects the dispersed cells from killing by neutrophils through eDNA degradation (Liu et al., 2017). However, the interaction between the host immune system and different bacterial physiological states (biofilm and planktonic) during the lifespan requires further investigations.

Neutrophils, the first line of innate immune defense, play an important role in controlling a bacterial challenge (Rosales et al., 2016; Kraus and Gruber, 2021). Neutrophils employed two widely known killing mechanism, receptor-mediated capture and phagocytosis, when facing pathogens (Castanheira and Kubes, 2019; Chen et al., 2021). Recently, a new antibacterial mechanism, known as Neutrophil Extracellular Traps (NETs), has been identified and has generated much enthusiasm (Papayannopoulos, 2018). NETs are large, web-like structures composed of extracellular DNA associated with antimicrobial proteins such as histone, calprotectin, and serine proteases, which are assembled on the scaffold of decondensed chromatin (Brinkmann et al., 2004; Wartha et al., 2007). They can trap, neutralize, and kill various microbes, thus preventing microorganism dissemination and playing a crucial role in the innate immune response (Brinkmann et al., 2004; Beiter et al., 2006; Branzk et al., 2014).

Previous studies have proposed that when pathogens are too large to be phagocytosed, NETs will be released to prevent pathogen dissemination and kill organisms (Branzk et al., 2014). Planktonic and biofilm pathogens have specific virulomes and may interact with the host differently. Planktonic bacteria are small and could be phagocytosed easily, while biofilms are a sessile impenetrable community that may potentially induce NETs formation. Well-documented studies have shown that *Pseudomonas aeruginosa* and *Staphylococcus aureus* biofilms could elicit the formation of NETs by neutrophils; in response, NETs amplify the expansion of the biofilms (Pieterse et al., 2016; Bhattacharya et al., 2018). However, neutrophils seem to fail to release NETs when faced with biofilms formed by *Candida albicans*, *Candida glabrata*, and *Streptococcus pneumoniae* compared with their planktonic counterparts (Thornton et al., 2013; Johnson et al., 2016; Johnson et al., 2017). Our poor understanding of the means through which neutrophils discriminatively handle the bacterial biofilm and planktonic lifestyle may help to explain why bacterial infections are difficult to eradicate. In this sense, we previously reported that the dispersed cells of *S. mutans* trigger the formation of NETs and assist in the clearance of bacteria (Liu et al., 2017), though it remained a mystery whether there is a distinguishing NETs reaction of neutrophils upon encounter with biofilm and planktonic *S. mutans* during the bacteria lifespan.

This study aimed to investigate the NETs-releasing capabilities of neutrophils challenged by *S. mutans*, grown in either the biofilm or planktonic state, and the resulting survival of the immune attack. The inhibition of the immune function of neutrophils intrigued us to explore the molecular mechanism involved in facilitating subsequent escape of different lifestyle cells from NETs entrapment.

## Material and methods

### *S. mutans* planktonic and biofilm growth

The bacterial strains used in this study were *S. mutans* UA159. Regarding biofilm formation, overnight culture of *S. mutans* was resuspended in BHI-1% sucrose (1:20), and 200 µL of the suspension was added to each well of 96-well plates followed by incubation for 4 h and 24 h at 37°C, unless otherwise specified. To disrupt the biofilm architecture in some experiments, biofilms were mechanically dispersed by gently pipetting, mimicking the disruption of the biofilm (Johnson et al., 2016). Regarding planktonic cells preparation, overnight culture of *S. mutans* was resuspended in BHI (1:20) and incubated at 37°C to the mid-log phases, and the cells were washed twice with PBS. To determine an equivalent burden of planktonic cells and biofilm, an XTT (2,3-Bis-(2-Methoxy-4-Nitro-5-Sulfophenyl)-2H-Tetrazolium-5- Carboxanilide) reduction assay was performed (Nett et al., 2011). A burden of 1.5 × 10^8^ planktonic cells per well was found to be equivalent to the biofilm burden (data not shown). Thus, this number of planktonic cells was used in a co-culture study to compare the response of neutrophils to the *S. mutans* biofilm, dispersed biofilm, and planktonic cells.

### Human neutrophils isolation

Human venous blood was obtained from volunteer donors after obtaining a written informed consent through a protocol approved by Guanghua School of Stomatology, Hospital of Stomatology, Sun Yat-sen University. Primary human neutrophils were purified using a Ficoll-Hypaque gradient and erythrocyte lysis as described elsewhere (Nauseef, 2007). The neutrophil preparations were > 95% pure by phase-contrast microscopy and were used within 30 min of purification. Studies with neutrophils were performed in RPMI 1640 with the addition of 10% FBS and incubated at 37°C with 5% CO_2_. Replicate experiments used neutrophils from different donors.

### Sytox Green assays

NET formation was measured by fluorescence Sytox Green assay as described previously (Branzk et al., 2014; Juneau et al., 2015). Briefly, *S. mutans* biofilms were grown in 96-well black plates for 4 h and 24 h, and neutrophils were added to a final concentration of 3 × 10^5^ cells per well for incubation. For a subset of experiments, cells were stimulated either with 100 nM PMA (Phorbol 12-myristate 13-acetate; Sigma) or *S. mutans* planktonic cells (equivalent burden to biofilm cells) or 24-h dispersed biofilm or left untreated. Cells were lysed with 1% Triton X-100 as a 100% control. Cell-impermeable Sytox Green DNA-binding dye (Life Technologies, Eugene, OR, USA) was added at a final concentration of 5 μM, and fluorescence was monitored every 30 min for a period of 6 h in a POLARstar Omega microplate reader (BMG Labtech, Ortenberg, Germany). Besides, the corresponding bacteria incubating in media without neutrophils was employed to eliminate the background fluorescence and purified neutrophils incubated in media was considered as negative control in our study. Then the relative fluorescence value of sample was calculated as percentage of DNA fluorescence compared with a Triton X-100 lysis control, which was reported in our previously study (Liu et al., 2017).

### Reactive oxygen species (ROS) measurement

For measurement of neutrophil ROS production, a modified oxidative stress assay was performed (Seper et al., 2013). Briefly, neutrophils were pre-stained with the fluorescent dye 2,7-dichlorodi- hydrofluorescein diacetate (DCFH-DA) in RPMI 1640 for 10 min at room temperature in the dark. The stained cells were added to *S. mutans* 4 h and 24 h biofilms, 24 h dispersed biofilms, and planktonic cells in 96-well black plates to a final concentration of 3 × 10^5^ neutrophils per well. Fluorescence (excitation 495 nm; emission 527 nm) was recorded every 30 min for 3 h, and data were shown for 2.5 h using a fluorescence reader. Results were calculated by subtracting fluorescence at time 0 and were expressed as relative fluorescence units.

### Visualization of NETs formation

To qualitatively measure NETs formation, a modified immunofluorescent imaging was performed (Johnson et al., 2016). *S. mutans* biofilms (4 h and 24 h) were grown in glass coverslip bottom petri dishes (MatTek, Ashland, Ma). After 4 h and 24 h of growth, biofilms were washed three times with DPBS, and neutrophil cell suspension in RPMI 1640 with 10% FBS was added at a concentration of 2 × 10^6^ cells/mL, followed by centrifugation at 800 g for 10 min to obtain the neutrophils in contact with the biofilms formed. Overnight *S. mutans* planktonic cells were co-cultured with neutrophils at a multiplicity of infection of 200 (MOI = 200). Neutrophils treated with 100 nM PMA were included as a positive control. After incubation for 4 h, cells on the cover slides were fixed with 4% paraformaldehyde for 10 min, followed by treatment with 1% Triton X-100 for 10 min and incubation with antibody blocking buffer (5% bovine serum albumin, Sigma) for 1 h. All steps were performed very gently to preserve the NETs. The cells were then incubated with the primary antibody anti-neutrophil elastase (anti-NE; abcam68672) and a secondary antibody conjugated to HRP (abcam6721) to visualize the NETs. The NETs DNA backbone was detected with DAPI. Specimens were mounted in an anti-fade fluorescence medium (Life Invitrogen) and observed with an oil immersion objective fluorescent microscope (Olympus FV3000). Images were processed using ImageJ.

### Assessment of the anti-biofilm capacity of neutrophils

Viability of *S. mutans* biofilm following co-culture with neutrophils was determined by an XTT assay, which measures residual metabolic activity in biofilms after exposure to neutrophils (Katragkou et al., 2010; Nett et al., 2011). Following a 24-h growth period, *S. mutans* biofilms in 96-well plates were washed with DPBS, and neutrophils cell suspension in RPMI 1640 with 10% FBS were added to a final concentration of 1.5 × 10^5^ cells/well. For a subset of experiments, neutrophils were treated with the same burden of planktonic *S. mutans* cells (MOI = 200). Following a 4-h incubation, neutrophils were lysed for 20 min at 37°C in TritonX-100 (0.03%) with 50 RPM agitations. Then, 90 μL of 9:1 XTT working solution (0.75 mg/mL XTT in PBS with 2% glucose: phenazine methosulfate 0.32 mg/mL in ddH2O) was then added to each well. After incubation for 30 mins, the plates were centrifuged at 1200 × g to pellet cells, and the supernatants (110 μL) of each well were transferred to a new clean plate for absorption reading at 492 nm. To determine the percentage killing, values were compared to wells without neutrophils after subtraction of the baseline absorbance. A subexperiment was performed to examine the anti-biofilm capacity of PMA pre-stimulated neutrophils. Briefly, neutrophils were pre-stimulate with PMA for 90min. After incubation, the medium was carefully replaced with serum-free RPMI 1640 and incubated further for 20min before infection with bacteria. (Brinkmann et al., 2004; Urban et al., 2006; Johnson et al., 2017).

### Scanning electron microscopy

*S. mutans* resuspended in BHI-1% sucrose at 1.5 × 10^6^ cells/mL was added to poly-L-lysine coated coverslips (13 mm, Termanox plastic for cell culture) and allowed to adhere for 30 min at 30°C. After removal of media and non-adherent cells, 1 mL of BHI-1% sucrose was added. Biofilms were propagated for 24 h at 37°C and washed twice with DPBS. Neutrophils (5 × 10^5^) were added to the biofilm coverslips for 2 h, 4 h, or 6 h, washed gently with DPBS, and prepared for scanning electron microscopy, as described previously. For studies utilizing planktonic organisms, a similar burden of planktonic *S. mutans* was added to the coverslip prior to the addition of neutrophils. PMA was employed as a positive control.

### Western blot

Cells were collected and lysed in lysis buffer for 30 min at 4°C. Then, lysates were centrifuged at 12,000 RPM at 4°C. Bio-Rad protein assay (Bio-Rad Lab, Hercules, CA) was employed to measure the protein concentration according to the manufacturer’s instructions. Briefly, 30–50 μg protein were fractioned in SDS-PAGE and then transferred to a polyvinylidene fluoride (PVDF, Millipore, Bedford, MA) membrane. The PVDF membrane was blocked with 5% skimmed milk at room temperature for 1–2 h and then incubated with specific primary antibodies overnight at 4°C. After thorough washing with PBST, horseradish peroxidase-labeled secondary antibodies were diluted and incubated for 1 h at room temperature. The immune complexes were finally visualized under the enhanced chemiluminescence (ECL) detection system. For western blot analysis, rabbit anti-human Rac-1, Rac-2, Dectin-1, and CARD-9 antibodies were purchased from Cell Signaling Technology (Danvers, MA). Signal intensities were analyzed by densitometry using ImageJ software, and values were standardized to the loading control.

### Bacterial survival in the circulation and pathological changes *in vivo*

The animal study was carried out according to the guidelines of the Animal Welfare Council of China and approved by the Ethical Committee for Animal Experiments of Sun Yat-sen University Cancer Center, China (L102012022060I). Balb/C mice (6–7 weeks) were anesthetized and subcutaneously challenged with planktonic and biofilm *S. mutans* at 1.5 × 10^8^ cells/mL as described previously (Walker et al., 2020). To further evaluate the effect of NETs, DNase I was injected with planktonic and biofilm *S. mutans* simultaneously, and at 12 h post-infection, DNase I was challenged again, while PBS challenge was employed as a control. To estimate the survival of *S. mutans* in the circulation, blood was collected from the eyeball at 4 h, 8 h, and 24 h post-infection and then serially diluted and streaked on BHI agar plates for CFU counting. Besides, at 24 h after infection, the mice were euthanized and the heart, liver, spleen, and lungs were collected and processed for hematoxylin-eosin staining to observe the pathological changes *in vivo*. Five mice were enrolled per groups and the experiment was repeat three times at different days.

### Statistics

Experiments were carried out at least 3 times using neutrophils from different donors on different days. Data was collected and analyzed with SPSS 24.0 software. All data were expressed as mean ± SD. Statistical analyses were performed by Student’s t-test (two-tailed) and one-way ANOVA with Bonferroni multiple comparisons *post-hoc* test. A *p*-value < 0.05 was considered statistically significant.

## Results

### As the lifestyle of *S. mutans* shifted from planktonic to biofilm, the of NETs-releasing capability changed from enhanced to diminished accordingly

**Figure 1:**
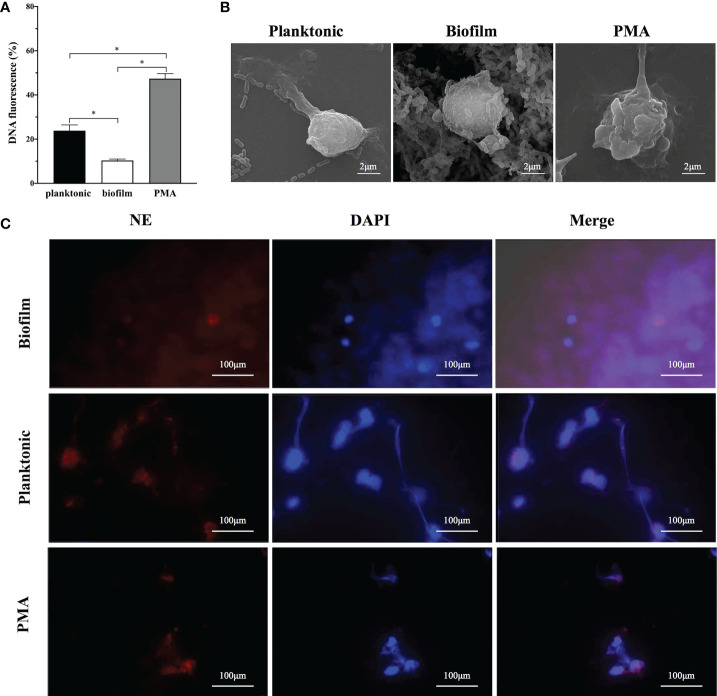
The capability of NETs releases according to different lifestyles of *S. mutans*. **(A)** Planktonic and biofilm *S. mutans* were co-cultured with neutrophils for 4h and NETs release was estimated by Sytox green assay. PMA was employed as the positive control. After 4h incubation, the fluorescence reached at 23 and 10% of the total DNA when stimulated with planktonic and biofilm *S. mutans* respectively. Data were depicted as mean ± SD (n=6). Statistical analysis was performed using a Student’s t-test with *p*<0.05. *p<0.05 **(B)** Representative scanning electron microscopy image of neutrophils after 4 h co-culture with *S. mutans* biofilm and its planktonic counterpart. A web-like structure of NETs coating the planktonic *S. mutans* was observed. In contrast, neutrophils exposed to biofilm showed no significant morphological changes, with no extruding web-like structure (scale bar: 2 μm). **(C)** Visualization of NETs formation by immunofluorescence imaging. Neutrophils were stimulated with PMA, *S. mutans* planktonic, and biofilm for 4h. The pictures from the left to right were labeled with the following dyes:NE with HRP conjugated (red), DNA with DAPI (blue), and an overlay of the first two pictures using Olympus FV3000 software. (scale bar: 100 μm).

Sytox Green, a cell-impermeable dye binding to DNA and emitting fluorescence signals, was used to quantify NETs release stimulated by different physiological states of *S. mutans*. When co-incubated with human neutrophils for 4 h, both planktonic and biofilm of S*. mutans* triggered fluorescence, which indicated the production of NETs (**Figure 1**). After 4h incubation, the fluorescence reached at 23 and 10% of the total DNA when stimulated with planktonic and biofilm *S. mutans* respectively, indicating that NETs releasing was significantly inhibited by *S. mutans* biofilm (**Figure 1A**).

To further identify whether the sytox green staining of free DNA veritably represents NETs release, scanning electron microscopy was employed to visualize neutrophils-*S. mutans* interactions (**Figure 1B**). After 4 h co-culture, crinkled neutrophils with extruding web-like structure could be observed either treated with PMA or planktonic *S. mutans*. In contrast, when challenged with *S. mutans* biofilm, the morphology of neutrophils remained intact, and barely a web-like structure was noticed. Immunofluorescence imaging was employed to analyze the presentation of elastase (red) and neutrophils (blue) simultaneously (**Figure 1C**). Upon co-culture with planktonic *S. mutans* and PMA, neutrophils exhibited web-like structures, which was consistent with the result of SEM. However, web-like structures were rarely visualized when exposed to *S. mutans* biofilm.

### The shifting of NETs formation over the whole bacterial lifespan was time-dependent and integrity- independent

**Figure 2:**
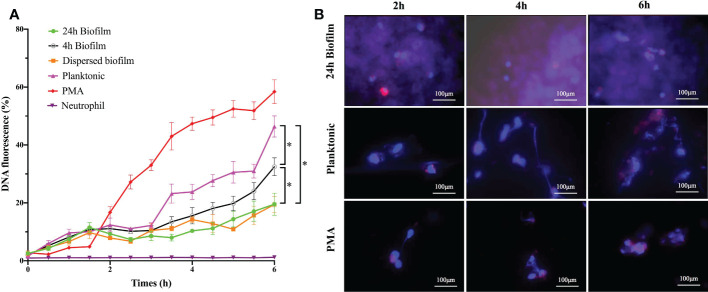
The shifting of NETs formation over the whole bacterial lifespan was time-dependent. **(A)** Kinetics of NETs release to different lifestyles of *S. mutans* (4h-biofim, 24h-biofilm, dispersed biofilm and planktonic) and PMA positive control during 6h co-culture period. NETs releases were quantified with a Sytox Green fluorescence assay. Neutrophils alone was served as the negative control. Data were presented as percentage of DNA fluorescence compared with a TritonX-100 lysis control (100%). Compared with the PMA positive stimuli, planktonic *S. mutans* generated high fluorescence, indicating NETs formation. In contrast, the 24-h biofilm and dispersed biofilm did not produce fluorescence. Besides, 4-h pre-mature biofilm did elicit NETs at a level lower than planktonic but higher than the mature biofilm. Data were depicted as mean ± SD (n=6). Statistical analysis was performed using a one-way ANOVA following Bonferroni multiple comparisons *post-hoc* test with *p*<0.05. *p<0.05 **(B)** Kinetics of NETs release visualized by immunofluorescence imaging. Neutrophils were stimulated with PMA, planktonic, and biofilm *S. mutans* for 2h, 4h and 6h respectively. As indicated by immunofluorescence staining, planktonic *S. mutans* elicited more NETs release with increasing co-culture time. The pattern of NETs release to planktonic *S. mutans* paralleled that of positive PMA stimulation. Staining of NE was barely observed even after 6 h of co-culture with the *S. mutans* biofilm. (scale bar: 100 μm).

Prior studies have demonstrated that the degree of NETs release to bacteria varies in their timing of release, dependence on reactive oxygen species (ROS), bacterial killing capacity, and so on. To further explore NETs release to different physiological state of *S. mutans*, we performed several complementary experiments to explore the kinetics of NETs formation upon contact with *S. mutans* immature biofilm (4 h biofilm), mature biofilm (24 h biofilm), and planktonic counterpart. PMA, a potent stimulus for NETs formation, was used as the positive control. As indicated by the Sytox Green assay, the NETs level increased over time with a lag-phase of 3 h for *S. mutans* planktonic and 4h biofilm stimuli, while only 1.5 h lag-phase was observed in PMA treatment (**Figure 2A**). Planktonic *S. mutans* and 4 h immature biofilm shared a similar NETs releasing pattern, which was different from the one of 24 h mature biofilm and dispersed biofilm. After 6h of co-incubation, the fluorescence of NETs increased over time and reached at about 46, 32 and 19% of the total DNA when stimulated with *S. mutans* planktonic, 4 h biofilm and 24 h biofilm, respectively(**Figure 2A**). Besides, the early elevation of free DNA in response to planktonic *S. mutans* indicated the involvement of rapid NETosis.

**Figure 3:**
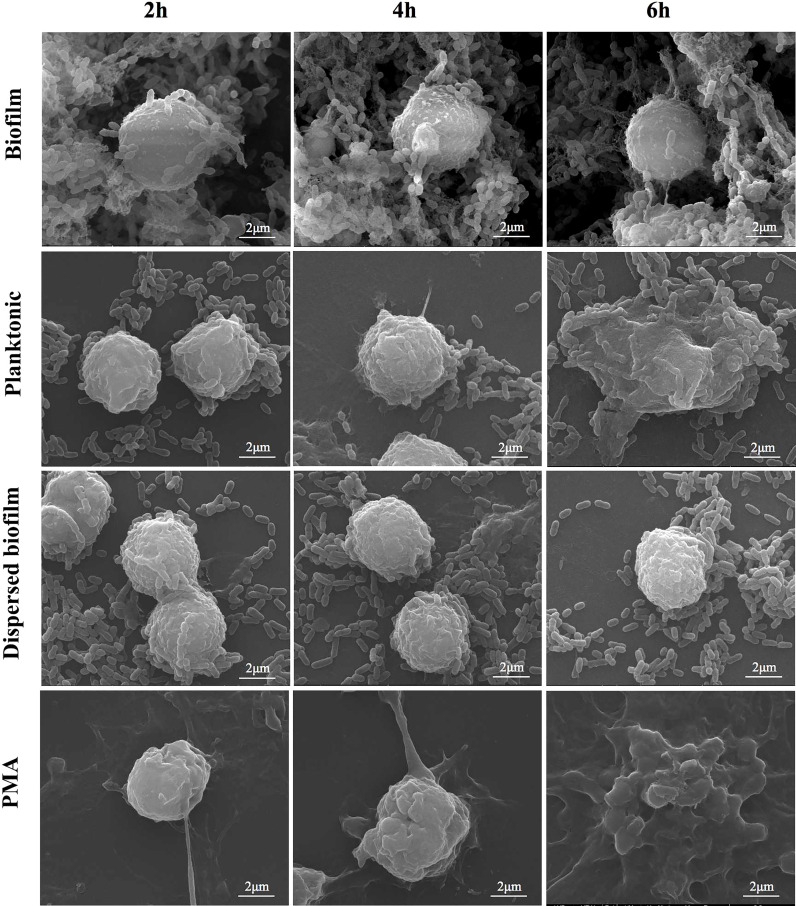
Observation of dynamic release of NETs. Neutrophils interactions with different lifestyles of *S. mutans* (planktonic, biofilm and dispersed biofilm) were observed under scanning electron microscopy at 2h, 4h and 6h co-culture timepoint. PMA was working as the positive stimuli. A web-like structure started to be released from neutrophils as early as 2 h after co-culture with PMA. For the planktonic stimuli, the web-like structure could be observed after 4 h of interaction. When the co-culture time was prolongated to 6 h, the natural round morphology of neutrophils all disappeared both for PMA and the planktonic stimuli. No significant morphological changes were noticed even after 6 h of co-culture with the 24-h mature biofilm or dispersed biofilm. (scale bar: 2 μm).

Scanning electron microscopy was utilized to monitor the morphological change occurring during neutrophils’ interaction with planktonic and biofilm *S. mutans*. Representative images are shown in **Figure 3** to illustrate the major changes among groups at different interaction time points. As a strong inducer of NETs, PMA triggers the changes in the morphology of neutrophils and the extruding of web-like structures over the co-culture time. Regarding planktonic *S. mutans*, the morphology of neutrophils changed from regular round to irregular crinkled and formless as the co-culture time increased. After 4 h of co-culture, we could clearly observe the web-like structure coating planktonic *S. mutans*. However, neither a change in morphology nor a web-like structure was rarely noticed even after 6 h of co-culture with biofilm *S. mutans*. To further evaluate NETs formation, we carried out fluorescent microscopy, revealing elastase (red) co-localization to the extracellular DNA associated with the neutrophils (blue) (**Figure 2B**). Immunofluorescent imaging showed that the pattern of NETs release to planktonic *S. mutans* paralleled that of the positive PMA stimulation, favoring more NETs formation along with increasing co-culture time. Scattered elastase was barely observed even after 6 h of co-culture with *S. mutans* biofilm, which was consistent with the finding of scanning electron microscopy. Taken together, these complementary time course studies revealed that extrusion of NETs from neutrophils was discriminatively affected by different physiological state of *S. mutans*, and the effect was time dependent.

The difference in the triggering of NETs formation by planktonic and biofilm *S. mutans* led to the investigation of the mechanism uncovering NETs formation to this pathogen. As widely accepted, a biofilm is a bacterial community encased by extracellular matrix; therefore, we speculate that the integrity of extracellular matrix may be a critical factor for the impairment of NETs formation. To investigate this, we physically dispersed the *S. mutans* biofilms and detected the neutrophils response. According to the Sytox Green assay, a similar NETs releasing pattern was shared by the dispersed biofilm and mature biofilm with a sustained low level of free DNA release during the whole co-culture period (**Figure 2A**). Scanning electron microscopy was also employed to monitor NETs release in response to the dispersed biofilm. Consistent with the result of the Sytox Green assay, neutrophils were observed to be round in appearance and barely had web-like structures (**Figure 3**). These combined results showed that the dispersed biofilm did not induce NETs formation, suggesting that the inhibition of NETs production by the *S. mutans* biofilm was integrity independent.

### *S. mutans* resisted neutrophils killing as planktonic single cells assembled into the biofilm community

**Figure 4:**
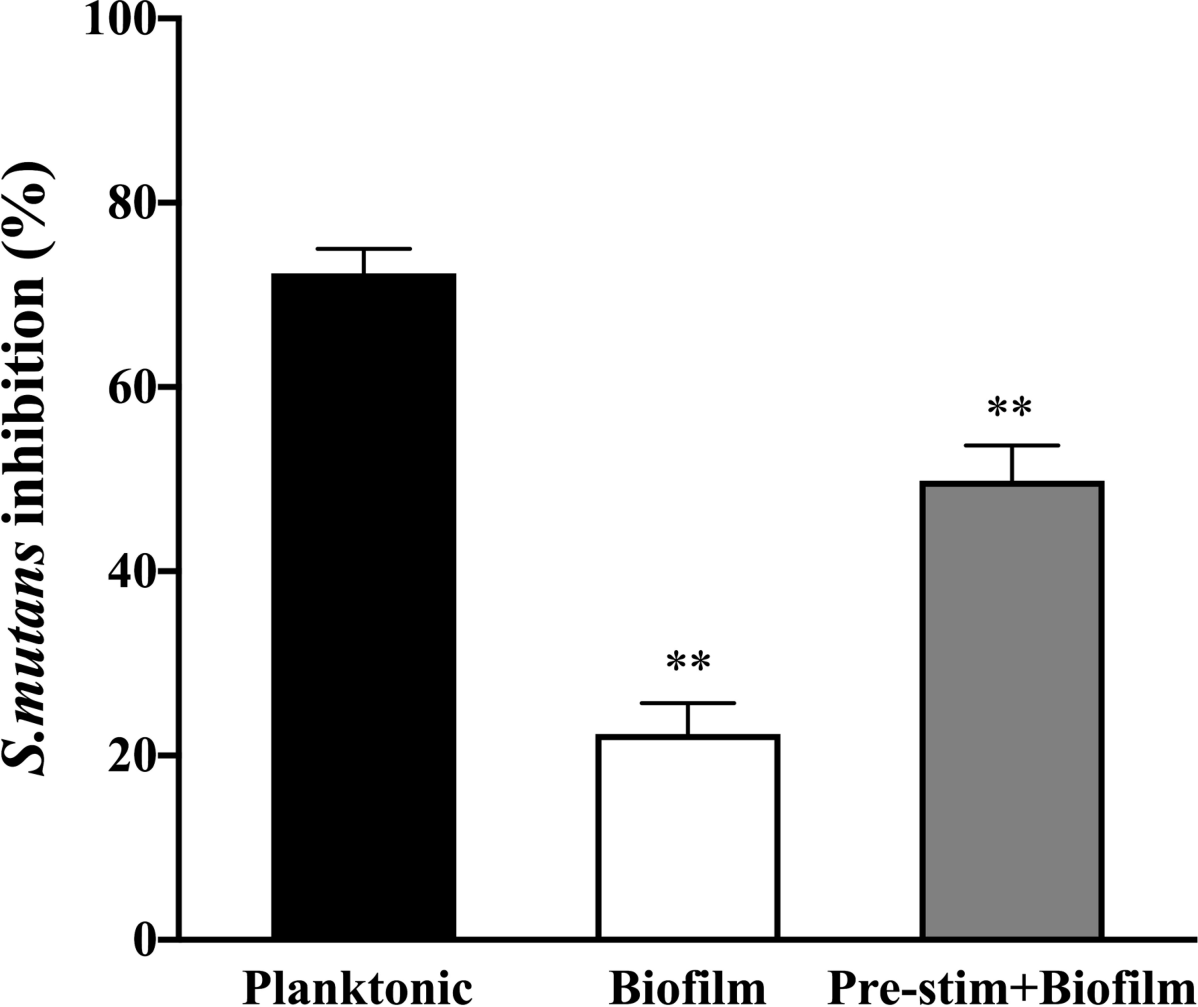
Survival advantage of *S. mutans* assembled into a biofilm community when facing neutrophils. Planktonic and biofilm *S. mutans* were co-cultured with neutrophils for 4h and bacterial burden were calculated by viable bacteria quantification assay. NETs were induced by incubation with PMA for 90min prior to infection with biofilm *S.mutans* as a subexperiment. Neutrophils exhibited strong killing activity against planktonic *S. mutans* (approximately 75%). However, when co-cultured with biofilm, neutrophils inhibited approximately 20% bacterial cells. Neutrophils pre-stimulated by PMA reversed the killing activity against biofilm. The results were depicted as mean ± SD (n=6). Statistical analysis was performed using a Student’s t-test with *p*<0.05. **p < 0.01.

To determine whether there is a survival advantage when planktonic bacteria assemble into the biofilm community, we measured the bacterial burden following co-culture with neutrophils. Viable bacterial burden was analyzed by XTT assay after lysis of neutrophils. While neutrophils exhibited a strong killing activity against planktonic *S. mutans* (approximately 75%), the bacterial inhibition of the biofilm co-cultured with neutrophils was only approximately 20% (**Figure 4**). Interestingly, the degree of biofilm killing was reversed by adding pre-stimulated neutrophils, indicating that neutrophils were effective against biofilm cells when pre-treated by PMA to allow the release of NETs. Together, impaired NETs formation protects the *S. mutans* biofilm to survive neutrophils attack.

### The ROS-dependent mechanism involved in NETs release stimulated by *S. mutans* evolved from individual bacteria to the biofilm community

**Figure 5:**
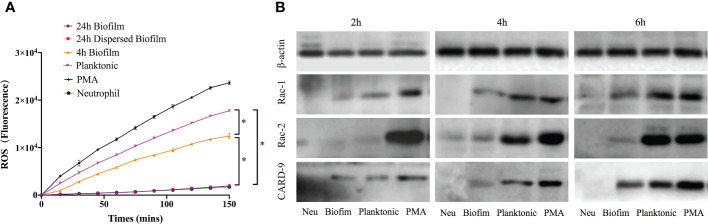
ROS-dependent pathway involved in NETs release stimulated by *S. mutans*. **(A)** ROS production by neutrophils exposed to *S. mutans*. Production of ROS in response to *S. mutans* was measured by fluorescence after neutrophils were pre-stained with DCFH-DA and co-cultured with different physiological state of bacteria over 150min. ROS production after exposure to 24-h biofilm was compared to that of neutrophils control or planktonic cells or 4 h biofilm or 24 h dispersed biofilm or PMA positive control at each time point using a one-way ANOVA following Bonferroni multiple comparisons *post-hoc* test with *p* < 0.05 (n=6), SEM shown. **(B)** The protein level of Rac-1, Rac-2, and CARD-9 were analyzed using Western Blot. Neutrophils were co-cultured with planktonic and biofilm *S. mutans* for 4h. PMA and neutrophils were served as positive and negative control, respectively. The expression levels of Rac-1, Rac-2, and CARD-9 were determined by Western Blotting. Expression of Rac-1 and Card-9 were significantly upregulated in a time-dependent manner when exposed to planktonic *S. mutans*. However, the relative expression of Rac-1, Rac-2, and CARD-9 remained at a low level when challenged with biofilm *S. mutans*. Data were representative of three separate experiments.

Prior investigations have proven that both ROS-dependent and -independent pathways are highly required for NETs formation in response to pathogens. The generation of ROS by neutrophils in response to *S. mutans* indicated the involvement of a ROS-dependent pathway for NETs formation (**Figure 5A**). The ROS level increased significantly when bacteria were presented in the planktonic state, almost 75% of the PMA positive control. When the bacteria cells started to aggregate and formed pre-mature biofilms, the ROS level decreased suddenly. As the biofilm evolved into a mature community, the production of ROS dropped steeply to the baseline level. Besides, the dispersed biofilm had the same ROS-releasing pattern as the mature biofilm.

ROS production was partially mediated by NADPH oxidase activation. Expression levels of several proteins related to the NADPH-oxidase pathway in neutrophils were determined by western blotting. As shown in **Figure 5B**, the protein expression of Rac-1 and CARD-9 of neutrophils stimulated by planktonic *S. mutans* were significantly upregulated (p < 0.05) in a time-dependent manner. However, the Rac-2 expression remained unchanged over the 4 h co-culture period. Regarding the response to biofilm *S. mutans*, the relative expression of Rac-1, Rac-2, and CARD-9 remained at a low level in a time-dependent manner. Together, these findings indicated that as bacterial cells aggregate and function as a biofilm community, they would inhibit NETs formation through a ROS-dependent mechanism involving the NADPH-oxidase pathway.

### *In vivo* reaction of neutrophils challenged with planktonic and biofilm *S. mutans*

**Figure 6:**
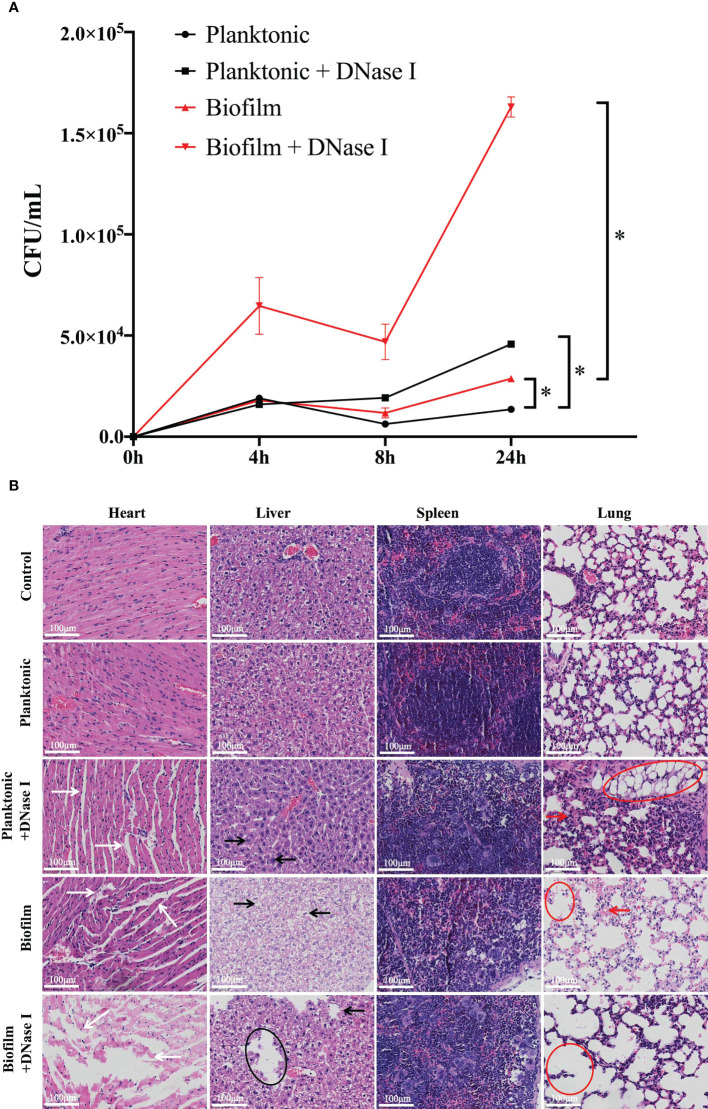
*In vivo* reaction of neutrophils challenged with different states of *S. mutans*. **(A)** A comparison of bacterial burden isolated from the mice blood post-infection between planktonic and biofilm *S. mutans* was shown. At 8 h and 24 h post-infection, more viable biofilm cells than planktonic cells could be detected. When DNase I was added to degrade NETs DNA, the bacterial cells in both challenge groups all displayed enhanced survival in blood, especially the biofilm cells. Comparison between planktonic and biofilm *S. mutans* with and without DNase I treatment was performed at each time point. The results were depicted as mean ± SD (n=5). Statistical analysis was performed using a one-way ANOVA following Bonferroni multiple comparisons *post-hoc* test with *p*<0.05. *p<0.05 **(B)** 6–7 weeks old Balb/C mice were infected with 1.5 × 10^8^ cells/mL of planktonic or biofilm *S. mutans* or were mock-infected with PBS. DNase I was applied with *S. mutans* simultaneously and 12h post-infection. Five mice were enrolled per treatment group. 24h after infection, the mice were euthanized and the histopathological characteristics were evaluated. Representative histopathological appearance of the heart, liver, spleen, and lung tissue following hematoxylin-eosin (H&E) staining in each group. The white arrows indicated a wide myocardial gap and blurred myofilament boundary in heart tissue. The black arrows and circles showed swollen liver cells with hepatic lipid droplets formation and steatosis. The red arrows and circles demonstrated abnormal structures of the lung alveoli with thickening alveolar walls and accumulation of alveolar vacuolation in lung specimen. (scale bar: 100 μm).

Through an *in vitro* study, we found that the biofilm state of *S. mutans* impedes the formation of NETs and survives neutrophils attack; therefore, we sought to determine whether there is a similar inhibition effect in an *in vivo* infection model. As indicated in **Figure 6A**, both viable planktonic and biofilm *S. mutans* cells decreased 8 h post-infection, which may be due to the immune response of the host. Besides, biofilm cells survived better than planktonic cells in the blood stream 24 h post-infection. When DNase I was added to destroy NETs, the survival of both planktonic and biofilm *S. mutans* was enhanced significantly. Notably, the viable bacteria in the biofilm were much more than that of planktonic cells when NETs were degraded by DNase I after infection, especially 24 h post-infection. These results suggested that the biofilm could enhance bacterial survival and avoid neutrophils attack in the host, especially when NETs were destroyed by DNase I.

Since biofilm *S. mutans* survive neutrophils attack *in vivo*, we further analyzed the pathological changes of the host. According to our research, the histological appearances caused by planktonic *S. mutans* were essentially the same as those in the control group. Specifically, myofibrils were orderly and organized in parallel rows, the lung tissue showed minor histopathological changes, and hepatocytes exhibited a regularly arranged morphology (**Figure 6B**). However, the histopathological changes induced by the biofilm were much more obvious, showing widen myocardial gap and blurred myofilament boundary, thicker alveolar septum and accumulation of alveolar vacuolation, and swollen liver cells with hepatic lipid droplets formation and steatosis. Markedly, when DNase I was added with either planktonic or biofilm *S. mutans* synchronously, a severely distorted structure with ruptured capillaries and large areas of necrosis with tissue collapse could be noticed everywhere. The response of the spleen was quite different in that no significant morphological changes were observed whether stimulated with biofilm or planktonic *S. mutans*.

## Discussion

Bacteria at different physiological states show different characteristics (Bjarnsholt et al., 2013). Before forming a biofilm, *S. mutans* exists as free-floating cells known as planktonic lifestyle, which could be easily eliminated by antibiotics and immune attack. The ability of *S. mutans* to persist in the oral cavity and disseminate throughout the host under certain favorable circumstances is associated with their capacity to form biofilms, which shelter *S. mutans* from a range of stresses and clearance mechanisms, thereby impeding the access of the immune system and antimicrobials (Huang et al., 2011; Scharnow et al., 2019). Two main questions remain unanswered to date: (i) how the host defense immune system reacts discriminatively to planktonic and biofilm *S. mutans* and (ii) whether *S. mutans* biofilm can be efficiently recognized and killed by immune cells. Neutrophils are the first reaction immune cells in the circulation to thrust a potent response to evasive pathogens and surveille host tissue, which plays a key role in innate immunity (Mantovani et al., 2011; Rosales et al., 2016; Kraus and Gruber, 2021). They can react to bacterial infection rapidly and eradicate invasive pathogens powerfully. It has been shown that biofilm formation by *A. fumigatus* resists neutrophils NETs killing compared with its planktonic counterpart (Lee et al., 2015), whereas *Staphylococcus aureus* growing as biofilms has developed the ability to avoid neutrophil killing through releasing Panton-Valentine leukocidin and ƴ-hemolysin AB (Bhattacharya et al., 2018).

In this study, we investigated the interaction of *S. mutans* with neutrophils, exploring the specific response by biofilm and planktonic bacteria. Our results showed that *S. mutans* mature biofilm interrupted NETs formation compared to their planktonic counterpart, whereas biofilms lacking a mature extracellular matrix (pre-mature biofilm) elicited low-level NETs release. Considering the dynamic development of the extracellular matrix enwrapping the biofilm cells closely, it is reasonable to conjecture that the inhibition NETs formation by the *S. mutans* biofilm was linked to the production of an extracellular matrix. Besides, the decrease in NETs correlated with the insusceptibility to neutrophils killing. Consistent with our results, Johnson CJ (Johnson et al., 2016) also found that *C. albicans* biofilm triggered negligible release of NETs compared to its planktonic counterpart, and the inhibition effect was dependent on an intact extracellular biofilm matrix. A similar result was also presented in the case of biofilms formed by other pathogenic bacteria such as *Streptococcus suis* serotype 2 and Candida spp., including *Candida glabrata* and *Candida parapsilosis* (Katragkou et al., 2011; Xie et al., 2012; Johnson et al., 2017; Ma et al., 2017). Furthermore, we found that neutrophils pre-stimulated by PMA, which produced NETs, were active against the biofilm. Although the biofilm inhibited NETs formation, NETs seemed to be an efficient mechanism to eliminate the *S. mutans* biofilms. Taken together, our results demonstrated that unlike the free-floating cells, biofilm formation conferred to *S. mutans* an enhanced ability to escape NETs recognition and killing, and the extracellular matrix of the biofilm may play a critical role in inhibiting NETs release, thus contributing to avoid an immune attack and providing a survival advantage.

To unravel the molecular mechanism behind different NETs reaction to either planktonic or biofilm *S. mutans*, ROS production and classical pathway activation were investigated, as these cascades were essential for NETs formation and immunity against pathogens. As indicated, biofilm formation conferred to *S. mutans* an enhanced ability to circumvent the early activation of this pathway by ROS-dependent mechanism, which was consistent with previous results obtained with Candida spp., for example, *Candida glabrata* and *Candida albicans* (Xie et al., 2012; Johnson et al., 2016; Johnson et al., 2017). NADPH oxidase in the ROS pathway has a critical role in NETs formation (Remijsen et al., 2011). Generally, ROS generated by NADPH oxidase stimulates myeloperoxidase to initiate the activation and translocation of NE to the nucleus, where NE proteolytically processes histones to trigger chromatin recondensation, and finally NETs release (Papayannopoulos et al., 2010; Papayannopoulos, 2018). Rac-1 (Ras-related C3 botulinum toxin substrate 1) is a small GTPase essential for the assembly and activation of NADPH oxidase. Rac-2, one of the subunits of NADPH oxidase, works as a cytosolic GTP-binding protein that regulates neutrophils oxidative burst (Knaus et al., 1992; Bu et al., 2021). CARD9, well known as a signaling adaptor protein, has been highly indicated in the activation of anti-pathogen immune responses and immune surveillance (Drummond et al., 2018). In the current study, Rac-1, Rac-2, and CARD-9 expression of neutrophils were suppressed by *S. mutans* biofilm, suggesting the involvement of the ROS-dependent NADPH pathway in NETs formation. Interestingly, a process of ROS-independent rapid NETs release has been monitored for planktonic *S. mutans* since NETs release was identified both by Sytox Green staining and scanning electron microscopy imaging at the very early co-culture time point (15 min-1 h), which has also been reported in *Staphylococcus aureus* (Bhattacharya et al., 2018).

Currently, however, the exact reaction of neutrophils facing invasive *S. mutans in vivo* remains unclear. Our blood survival assay suggested that *S. mutans* biofilm was better able to survive than their planktonic counterpart *in vivo*, which was consistent with the result of *Streptococcus suis* serotype 2 infection (Ma et al., 2017). When NETs DNA was degraded by DNase I, bacteria in the biofilm was protected from being killed and exhibited significantly enhanced survival in blood, which indicated that NETs in the blood stream may be a vital bactericidal mechanism in trapping, neutralizing, and killing biofilm bacteria. In addition, our histological results demonstrated that planktonic *S. mutans* cause slight pathological changes in tissues such as the heart, lung, liver, and spleen. Interestingly, our histological findings were inconsistent with the results of others in that whether *S. mutans* could induce significant histopathological changes or not *in vivo* animal study. For example, by infection with *S. mutans*, Velusamy et al. demonstrated the infiltrated with inflammatory cells and abnormal structures of the lung alveoli with thickening alveolar walls in lung tissues; prominent accumulation of lipids and macrovesicular appearance along with ballooning and vacuolization of hepatocytes and significant infiltration of inflammatory cells in liver, and splenomegaly and infiltrations of increasing inflammatory cells in spleen specimen (Kim et al., 2012; Hong et al., 2014; Naka et al., 2014; Velusamy et al., 2014; Naka et al., 2016; Yu et al., 2022). However, the duration of bacterial infection was much longer in those studies, and this helped to explain the inconsistent results partly. When challenged with *S. mutans* biofilm, the heart, lung, and liver all lost their basic architecture and displayed apparent histopathological changes including atrophy, distortion, and steatosis. Importantly, when NETs of neutrophils were destroyed by DNase I, more crushing damage could be observed with large areas of necrosis and tissue collapse. Together, these findings indicated that unlike planktonic bacteria, biofilm formation is a survival and an assault strategy employed by *S. mutans* to evade host immune system attack and induce ruinous destruction *in vivo*.

The data from this study clearly suggest that the extent of NETs response to the presence of bacteria is greatly dependent on whether it is a free-floating single cell or an aggregated bacterial community. A pronounced impairment in NETs release could be expected when bacteria assemble into a biofilm. However, as suggested by our study, mechanical disruption of the biofilm matrix did not reverse the inhibition phenotype or re-motivate NETs formation of planktonic cells to a similar level, which indicated that the extracellular biofilm matrix (ECM) may play an important role in the release of NETs. The ECM was composed of carbohydrates, lipids, proteins, nucleic acids, uronic acids, and so on, which formed the framework for the three-dimensional biofilm structure (Kassinger and van Hoek, 2020; Monticolo et al., 2020). Polysaccharides, mannan-glucan complex, and extracellular nucleic acids have been linked to the release of NETs in Candida spp (Zarnowski et al., 2014; Johnson et al., 2016; Smolarz et al., 2021). However, the exact role of the ECM employed by *S. mutans* in NETs immune evasion needs to be fully considered, and this work is currently being conducted in our lab. Besides, in the current investigation, we defined the bacterial physiological state according to the start time-point of co-culture. However, the bacterial phase changed during the co-culture period, which may alter the NETs reaction of neutrophils to various extents. Therefore, in future study, we should focus more on the real-time observation of dynamic NETs reaction with the changes of co-culture period.

**Figure 7:**
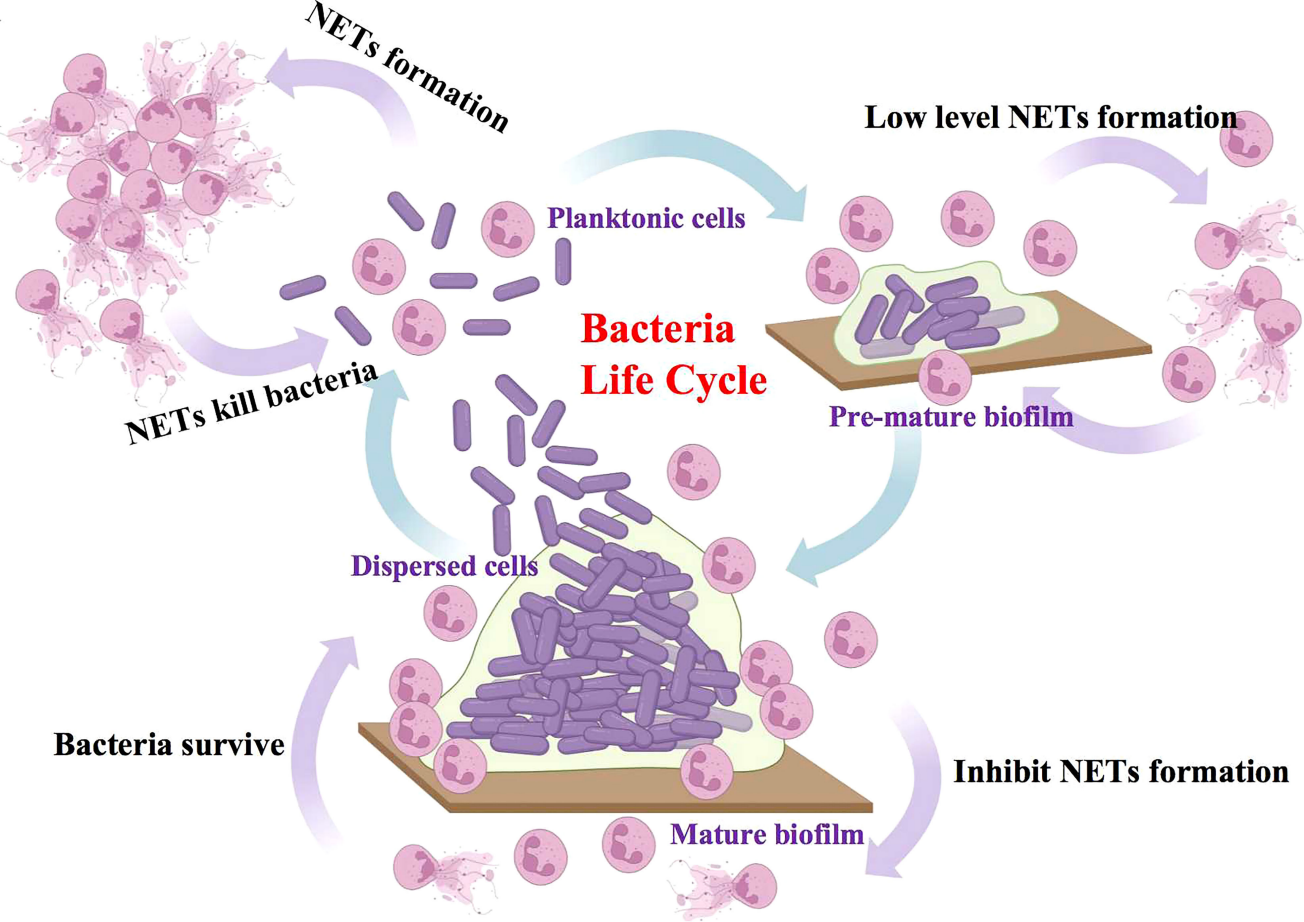
Proposed model of discriminatively NETs reaction of neutrophils to different physiological states of *S. mutans*. In the lifespan of *S. mutans*, the free-floating planktonic bacteria exhibited an active NETs response, which assisted in the elimination of bacteria in turn. However, as bacteria assembled into the mature biofilm community, they exhibited a reverse negative NETs response and as a result, protected bacteria from the immune attack.

In conclusion, our study investigated the discriminative reaction of neutrophils to different physiological states of *S. mutans*. In the lifespan of *S. mutans*, an active NETs response to free-floating planktonic cells could be expected. However, as bacteria assembled into a biofilm community, the response to NETs turned negative. When bacteria dispersed from the biofilm, they regained their active reaction to NETs at the same time (**Figure 7**). It is highly believed that the pronounced impairment of NETs formation by *S. mutans* biofilm was dependent on the oxidative burst generated through the NADPH pathway, which may account for the resistance to neutrophils killing. A better understanding of battles between NETs and *S. mutans* could point to potential new avenues for the development of immune-based treatments for invasive *S. mutans* infection.

## Data availability statement

The original contributions presented in the study are included in the article/supplementary material. Further inquiries can be directed to the corresponding author.

## Ethics statement

The studies involving human participants were reviewed and approved by Guanghua School of Stomatology, Hospital of Stomatology, Sun Yat-sen University. The patients/participants provided their written informed consent to participate in this study. The animal study was reviewed and approved by The Ethical Committee for Animal Experiments of Sun Yat-sen University Cancer Center, China (L102012022060I).

## Author contributions

Experiments were performed by the following authors: conceived and designed the experiments: LH and JL; performed the experiments: LH, WL, and YN; wrote the paper: LH; revised the manuscript: JL. The manuscript had been reviewed by all authors before submission. All authors contributed to the article and approved the submitted version.

## Funding

This work was supported by the Science and Technology Program of Guangzhou under grant 202102020148; the Natural Science Foundation of Guangdong Province under grant 2020A1515110064. Medical Scientific Research Foundation of Guangdong Province under grant A2022126.

## Conflict of interest

The authors declare that the research was conducted in the absence of any commercial or financial relationships that could be construed as a potential conflict of interest.

## Publisher’s note

All claims expressed in this article are solely those of the authors and do not necessarily represent those of their affiliated organizations, or those of the publisher, the editors and the reviewers. Any product that may be evaluated in this article, or claim that may be made by its manufacturer, is not guaranteed or endorsed by the publisher.
